# Integrated bioinformatics analysis of common molecular mechanisms and biomarkers in oral squamous cell carcinoma and periodontal disease

**DOI:** 10.1007/s12672-025-03728-0

**Published:** 2025-10-15

**Authors:** Lin Wu, Peng She, Chenguang Qiu, Hongtao Sun, Fanzhi Kong, Hong Wang, Yu Tian, Jun Yang, Zhenwei Mao

**Affiliations:** 1 Department of Stomatology, Affiliated People’s Hospital of Jiangsu University, Zhenjiang, China; 2 Department of Laboratory Medicine, Affiliated People’s Hospital of Jiangsu University, Zhenjiang, China; 3 Department of Laboratory Medicine, Fourth Affiliated People’s Hospital of Jiangsu University, Zhenjiang, China

**Keywords:** Oral squamous cell carcinoma, Periodontal disease, Machine learning, Immune infiltration, FNDC3B

## Abstract

**Background:**

Oral Squamous Cell Carcinoma (OSCC) and Periodontal Disease (PD) are two distinct yet interconnected conditions with complex molecular mechanisms. This study aimed to analyze transcriptomic data from both diseases to identify common and unique molecular features, uncover potential biomarkers, and explore therapeutic targets.

**Method:**

Transcriptomic data from OSCC and PD were analyzed using WGCNA to construct gene co-expression networks and identify disease-associated modules. Functional enrichment analyses were conducted to reveal shared biological pathways, particularly those related to extracellular matrix organization. Machine learning methods, including Lasso regression, Random Forest, and SVM-RFE, were applied to select key feature genes. The findings were validated using independent datasets. Immune infiltration analysis using CIBERSORT was performed to assess immune cell interactions, while single-cell RNA sequencing was employed to explore the cellular distribution and functional role of the identified biomarker FNDC3B.

**Result:**

WGCNA identified key gene modules associated with OSCC and PD, with functional enrichment analyses highlighting shared pathways involved in extracellular matrix organization. Machine learning approaches identified FNDC3B as a central gene in both diseases, and its differential expression was validated in independent datasets. Immune infiltration analysis demonstrated the involvement of FNDC3B in immune cell interactions. Single-cell RNA sequencing further revealed the enrichment of FNDC3B in specific cell types, providing deeper insights into its role in disease progression.

**Conclusion:**

This study elucidates the molecular similarities and differences between OSCC and PD, positioning FNDC3B as a critical biomarker for both conditions. The findings enhance our understanding of the shared and distinct mechanisms driving these diseases and provide a foundation for the development of targeted therapeutic strategies.

**Supplementary Information:**

The online version contains supplementary material available at 10.1007/s12672-025-03728-0.

## Introduction

Oral squamous cell carcinoma (OSCC) is one of the most common types of oral cancer worldwide, with high incidence and mortality rates [1, 2]. According to global cancer statistics, there are over 300,000 new cases of OSCC annually, and its incidence is increasing in some developing countries [3]. The etiology of OSCC is multifactorial, involving smoking, alcohol consumption, viral infections (such as human papillomavirus, HPV), and genetic susceptibility [4–6]. Despite recent advancements in the diagnosis and treatment of OSCC, the prognosis remains poor, with a five-year survival rate of approximately 50% [7]. Therefore, there is an urgent need to identify novel biomarkers and therapeutic targets to improve early diagnosis and treatment outcomes for OSCC.

Periodontal disease (PD) is a common chronic inflammatory disease that primarily affects the gingiva and periodontal supporting tissues [8, 9]. PD not only leads to tooth loss but is also associated with various systemic diseases, such as cardiovascular diseases, diabetes, and respiratory diseases [10–12]. In recent years, increasing evidence has suggested a potential link between PD and various cancers, including oral cancer [13, 14]. Studies have shown that patients with PD have a significantly increased risk of developing oral cancer, which may be attributed to the chronic inflammatory state and alterations in the immune system [15, 16]. The chronic inflammation associated with PD may create a favorable environment for cancer initiation and progression, promoting the proliferation and metastasis of cancer cells.

Based on the aforementioned background, we hypothesize that there may be common regulatory mechanisms and characteristic biomarkers between OSCC and PD. Previous studies have demonstrated that inflammatory factors such as IL-6, TNF-α, and IL-1β are significantly upregulated in both OSCC and PD, suggesting their potential roles in the pathogenesis of both diseases [17–20]. By conducting a comprehensive analysis of the transcriptomic data of OSCC and PD, we aim to identify these common regulatory mechanisms and characteristic biomarkers, thereby providing new insights into the diagnosis and treatment of both diseases. In particular, the roles of inflammatory responses and the immune system in these two diseases may represent a critical research direction.

Identifying common pathogenic factors between OSCC and PD is crucial. It not only aids in understanding the pathological mechanisms of both diseases but also lays the foundation for developing new diagnostic methods and therapeutic strategies. Moreover, discovering common biomarkers and regulatory mechanisms can enhance early detection and prevention, ultimately improving patient survival rates and quality of life. By thoroughly investigating these shared factors, we aim to achieve more precise treatments and personalized medical approaches in future clinical practice. We hypothesize that OSCC and PD share overlapping molecular and immune-related mechanisms—particularly involving inflammatory pathways and extracellular matrix remodeling—and that identifying these common biomarkers may provide novel targets for early diagnosis and therapeutic intervention.

## Methods

###  Data sources

**Fig. 1:**
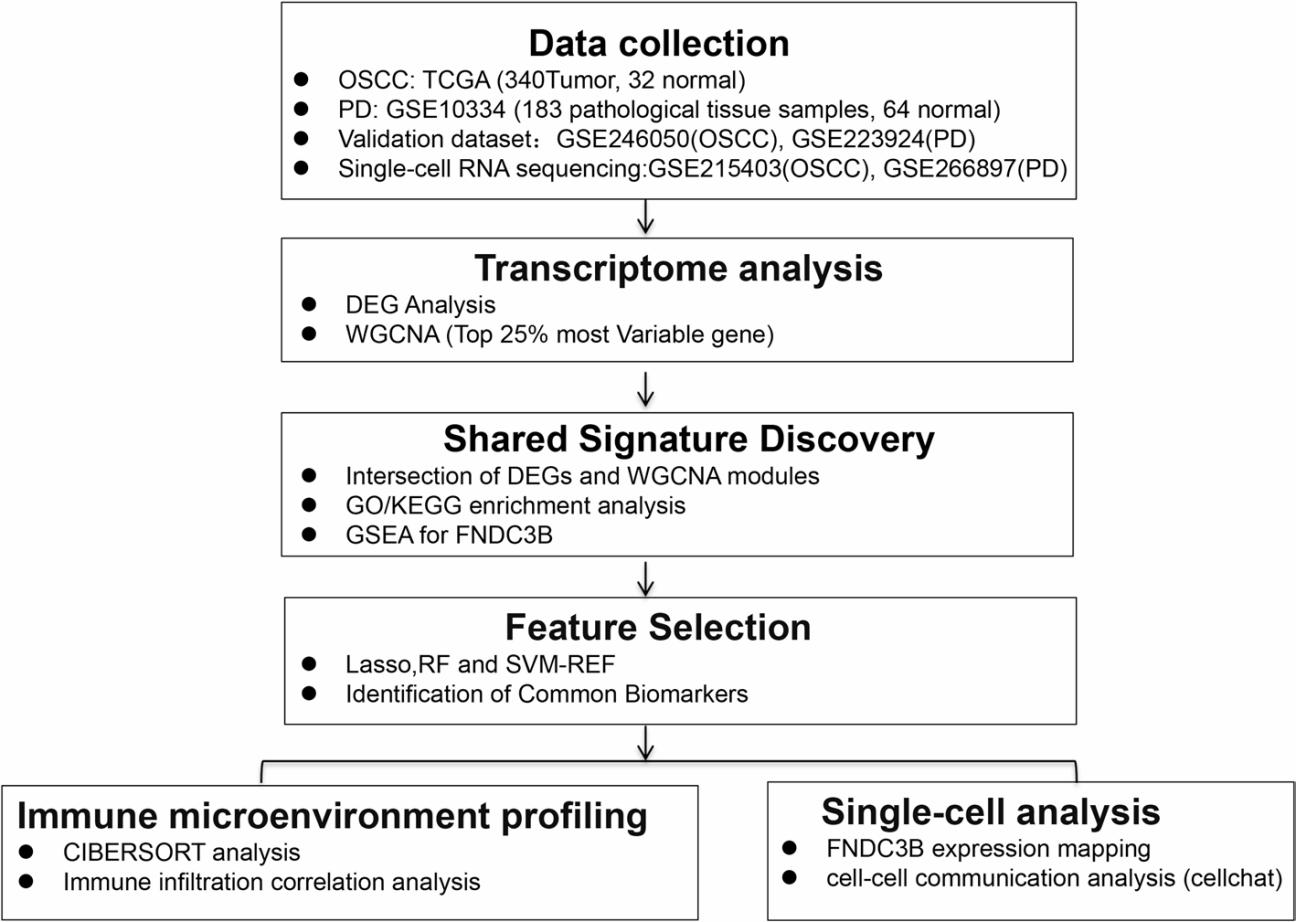
Schematic Overview of the Research Workflow

The OSCC dataset was obtained from The Cancer Genome Atlas (TCGA), consisting of 32 normal and 340 tumor samples, with expression data in TPM format. Detailed clinical features (e.g., age, gender, race, stage) and anatomical sites of tumor samples included in the TCGA-OSCC cohort are provided in Supplementary Table 1. The periodontal disease (PD) dataset GSE10334 was sourced from Gene Expression Omnibus (GEO) and is a microarray dataset comprising 64 normal and 183 pathological tissue samples. For validation, we used GSE246050 (TPM format, OSCC) and GSE223924 (TPM format, PD). Additionally, we used the GSE215403 dataset as the source of OSCC cell data, while PD single-cell data were derived from the GSE266897 dataset. The overall workflow is outlined in Fig. 1.

### Differential expression genes (DEGs) analysis

We first preprocessed the transcriptomic data of OSCC and PD, including data cleaning and normalization, to eliminate potential batch effects and ensure data consistency and reliability. Subsequently, DEG analysis was conducted for both datasets using the Limma package in R version 4.3.2. This analysis aimed to identify genes with significant differential expression between normal and pathological tissues, providing a robust data foundation for subsequent biological research and clinical applications by elucidating the molecular characteristics and alterations associated with OSCC and PD. To control for false positives due to multiple testing in transcriptome-wide analysis, p-values were adjusted using the Benjamini-Hochberg (FDR) method.

### Weighted gene co-expression network analysis (WGCNA)

In this study, we performed WGCNA on the transcriptomic data of OSCC and PD. To reduce noise and enhance computational efficiency, we selected the top 25% most variable genes for WGCNA. To identify and remove potential outliers, we first performed hierarchical clustering of all samples based on Euclidean distance and constructed a sample dendrogram. A static tree cutting approach was applied using the cutreeStatic function, with a height threshold of 20,000 to detect outlier samples. Samples not grouped into the main cluster were excluded from subsequent analysis. In this analysis, the clinical trait used for module-trait correlation was tissue status (normal vs. disease), which was matched to the expression data using sample identifiers. During sample clustering and heatmap analysis, we selected a power value range from 1 to 20. The optimal power value was determined by fitting a scatter plot of scale-free topology fit index against the power values and a scatter plot of mean connectivity against the power values. Using the optimal power value, we constructed the adjacency matrix and Topological Overlap Matrix (TOM). Genes were then clustered, dynamic modules were identified, and gene dendrograms and dynamic tree cut diagrams were plotted. We also clustered modules to identify similarities between them and plotted the module-gene heatmap. Finally, we analyzed the relationship between modules and trait data, generating a heatmap to illustrate the module-trait associations.

### Functional enrichment analysis

Gene Ontology (GO) and Kyoto Encyclopedia of Genes and Genomes (KEGG) enrichment analyses were conducted using the clusterProfiler package in R [21]. Gene symbols were first converted to Entrez IDs using the org.Hs.eg.db annotation package. GO analysis was performed with the enrichGO function, and KEGG analysis with enrichKEGG (organism = “hsa”), both using a p-value cutoff of 0.05. The results were visualized using bar and bubble plots generated with enrichplot and ggplot2.

### Machine learning

To identify characteristic genes for OSCC and PD, we employed a comprehensive approach utilizing multiple machine learning methods, including Lasso regression, random forest (RF), and support vector machine recursive feature elimination (SVM-RFE). First, Lasso regression, by incorporating an L1 regularization term, effectively reduces the number of features in high-dimensional data, thereby selecting key genes most relevant to the diseases. This method helps eliminate redundant variables while preserving critical genes essential for disease diagnosis. Next, the random forest algorithm assesses the importance of each gene by constructing multiple decision tree models. This method not only captures complex nonlinear relationships but also demonstrates strong resistance to noise. In the random forest model, the importance of each gene is determined by its contribution to the classification results, allowing for the precise selection of genes with significant discriminative power for OSCC and PD. Finally, we applied SVM-RFE, which recursively trains an SVM model and sequentially removes less important genes, ultimately identifying the most discriminative characteristic genes. SVM-RFE combines the powerful classification performance of SVM with a feature elimination strategy, optimizing the selection of genes for accurate classification. By integrating Lasso, random forest, and SVM-RFE, we ensured the robustness and consistency of the selected characteristic genes across different machine learning models.

### Nomogram analysis

To assess the risk of disease based on the expression of specific feature genes, we constructed a nomogram using a logistic regression model. The expression data were then transformed, and the sample group information was extracted to distinguish between different disease states. Subsequently, the data were prepared for modeling by using the datadist function, which sets up the distribution of the data for the logistic regression analysis. A logistic regression model (lrm) was built with the expression levels of selected genes as predictors of disease type. The nomogram was generated from this model using the nomogram function, which graphically represents the risk of OSCC and PD based on gene expression levels. The nomogram was calibrated with a function to predict the probability of disease occurrence across a range of values.

### Immune infiltration analysis

To analyze the immune infiltration levels in OSCC and PD samples, we utilized the CIBERSORT method. CIBERSORT employs a linear support vector regression model to estimate the relative proportions of 22 distinct immune cell types within each sample based on known immune cell expression signatures. Following this, we conducted a Spearman correlation analysis to assess the relationship between the expression levels of selected characteristic genes and the infiltration levels of these immune cell types.

### Gene set enrichment analysis (GSEA)

To explore the functional enrichment of the FNDC3B gene in OSCC and PD, we conducted GSEA. We began by processing the gene expression data, filtering out normal samples, and normalizing the data to ensure consistency. The samples were then divided into high and low expression groups based on the median expression level of FNDC3B. Samples were divided into high and low expression groups based on the median expression level of FNDC3B (4.6 for OSCC and 7.4 for PD). The log2 fold change between the high and low expression groups was calculated for each gene to generate a ranked gene list, which was then used as input for GSEA. The GSEA was performed using the KEGG gene sets (c2.cp.kegg.v7.4.symbols.gmt). Significantly enriched pathways were identified based on a p-value threshold of 0.05. This analysis allowed us to determine the biological pathways associated with differential expression of FNDC3B, providing insights into its potential role in OSCC and PD.

### Single-cell analysis

In this study, we performed single-cell RNA sequencing analysis to investigate the cellular heterogeneity within our samples. Initially, we conducted quality control by subsetting the merged Seurat object to retain cells with more than 500 detected genes, less than 20% mitochondrial gene expression, and more than 1000 RNA counts. Subsequently, the data were normalized using the NormalizeData function in Seurat, followed by identification of highly variable genes (FindVariableFeatures), scaling (ScaleData), and dimensionality reduction using principal component analysis (PCA). To correct for batch effects across multiple samples, we applied the RunHarmony function using the sample identity as the integration variable. Post-integration, we constructed a shared nearest neighbor graph using the Harmony-reduced dimensions and performed clustering with a resolution of 0.5. Finally, we identified marker genes for each cluster using the FindAllMarkers function with the Wilcoxon rank-sum test, considering only positive markers and applying a log fold change threshold of 0.25. These marker genes were subsequently used to annotate the cell types within each cluster, providing insights into the cellular composition and functional characteristics of the samples.

### Cell communication analysis

To analyze cell-cell communication in OSCC and PD using single-cell RNA sequencing data, we employed the CellChat package. First, we normalized the expression data and created a CellChat object, which included both the gene expression matrix and corresponding cell type annotations from the single-cell dataset. The human ligand-receptor interaction database from CellChat was used to facilitate downstream analysis. We identified overexpressed genes and interactions by extracting signaling genes from the expression matrix and mapping these interactions onto a protein-protein interaction network. The cell-cell communication probabilities were then computed to infer the likelihood of interactions between different cell types. Interactions involving cell groups with fewer than 10 cells were filtered out to ensure robust analysis. We subsequently aggregated the communication network data to summarize the number and strength of interactions between different cell types. These results were visualized in a circular plot format, depicting the interaction counts and weights, which represent the overall communication network’s complexity and intensity.

## Results

### Identification of differentially expressed genes in OSCC and PD

**Fig. 2:**
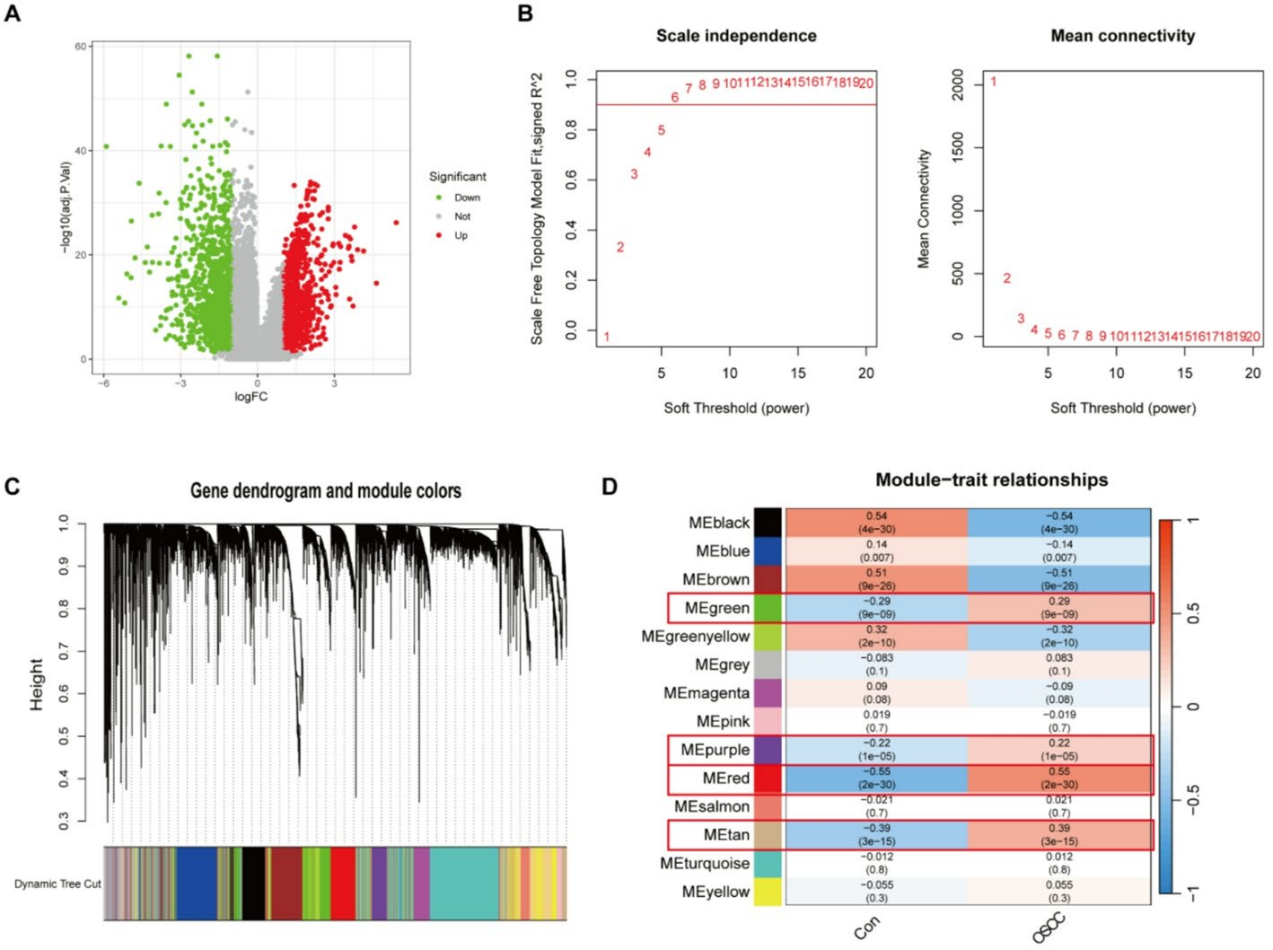
DEGs and WGCNA Analysis of OSCC. **A** Volcano plot showing differentially expressed genes (DEGs) between 340 OSCC and 32 normal tissue samples. Red and green dots indicate significantly upregulated and downregulated genes, respectively (|log2FC| >1, adjusted p-value < 0.05). **B** Soft-threshold power selection plot identifying power = 6 as optimal for constructing a scale-free co-expression network in OSCC using WGCNA. **C** Gene dendrogram and module assignment in OSCC based on hierarchical clustering and dynamic tree cut algorithm. **D** Module-trait relationship heatmap, depicting the Spearman correlation between each module and OSCC disease status. Four modules (MEgreen, MEpurple, MEred, MEtan) exhibited strong positive correlation with OSCC and were selected for further analysis

To identify differentially expressed genes (DEGs) in OSCC and PD, transcriptomic data from each condition were independently normalized and analyzed. For OSCC, a threshold of |Log2FC| >1 and adjusted p-value (FDR) < 0.05 was applied, resulting in the identification of 2,081 DEGs, among which 918 genes were upregulated and 1,163 were downregulated (Fig. 2A). In contrast, the analysis of PD utilized a more lenient threshold of |Log2FC| >0.5 with a adjusted p-value (FDR) < 0.05, leading to the discovery of 988 DEGs, with 588 upregulated and 400 downregulated (S. Figure 1A). These criteria were chosen to accommodate the distinct biological and transcriptomic characteristics of the two diseases, ensuring a robust identification of DEGs relevant to each pathology.

### Disease-correlated gene modules identified by co-expression network analysis

To further explore the gene networks associated with OSCC and PD, we performed WGCNA. This method allows for the identification of gene modules that correlate with disease traits. For OSCC, a soft-threshold power of 6 was selected (Fig. 2B), while a soft-threshold power of 16 was applied for PD (S. Figure 1B), reflecting the optimal scale-free topology for each dataset. The constructed co-expression networks classified genes into distinct modules, which were then correlated with clinical traits of interest (Fig. 2C and S. Figure 1C). Given the non-normal distribution commonly observed in gene expression data, we used Spearman correlation to assess module-trait relationships. Specifically, for OSCC, the MEgreen, MEpurple, MEred, and MEtan modules were selected for further analysis (Fig. 2D), while for PD, the MEblack, MEblue, MEgrey, and MEred modules were chosen (S. Figure 1D). These selected modules showed strong positive correlations with disease status, suggesting that their constituent genes may be involved in key pathological processes such as immune modulation, epithelial transformation, or tissue remodeling, which are commonly implicated in OSCC and PD.

### ECM remodeling and inflammatory pathway signatures in OSCC and PD

**Fig. 3:**
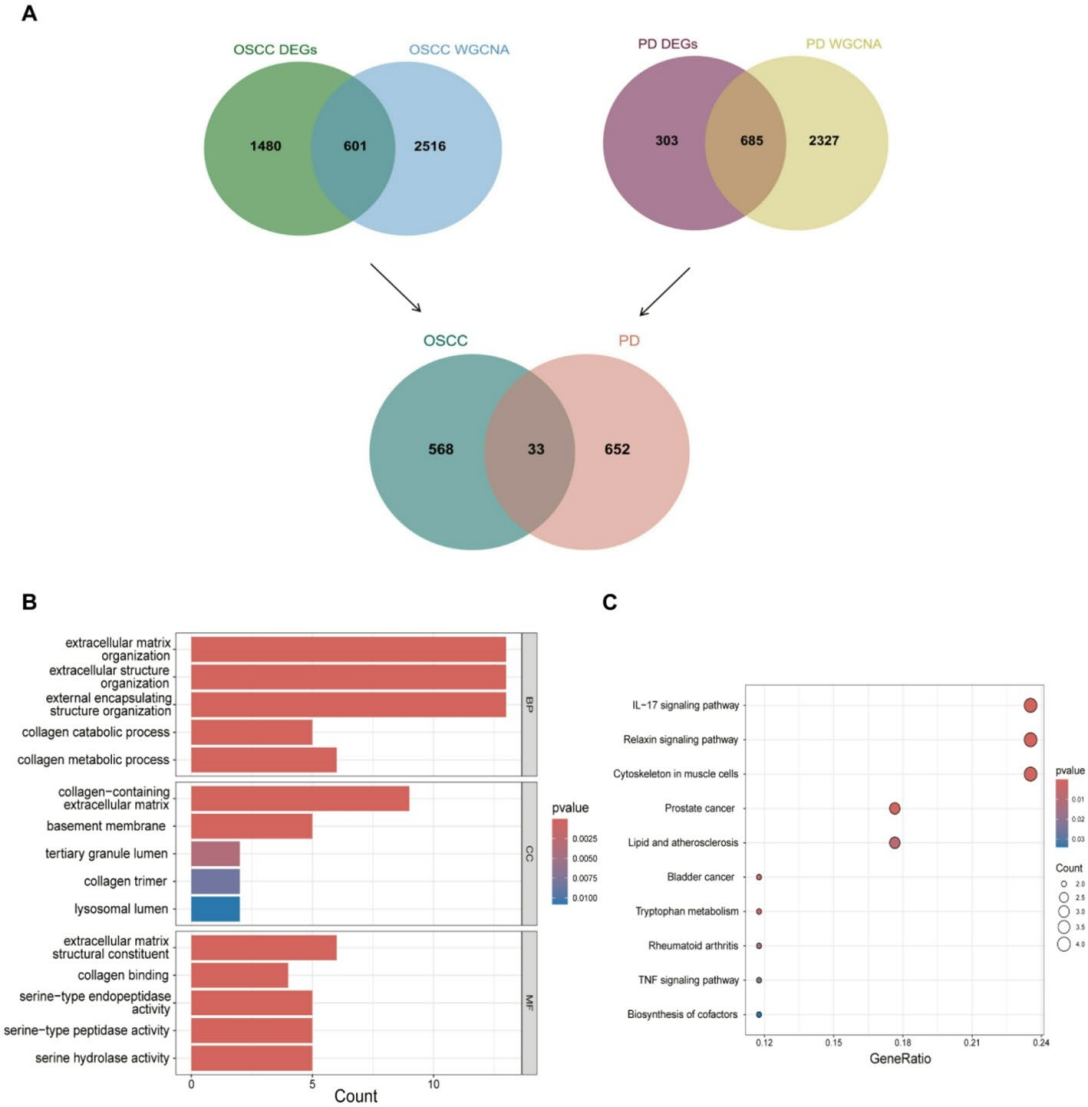
Functional Enrichment Analysis of Common Genes between OSCC and PD. ** A** Venn diagram showing 33 overlapping genes between DEGs and WGCNA module genes from both OSCC and PD datasets. **B** GO enrichment analysis results of the 33 common genes, highlighting enrichment in biological processes related to extracellular matrix organization and structural constituents. **C** KEGG pathway analysis of shared genes, revealing enrichment in IL-17 signaling and Relaxin signaling pathways, suggesting common inflammatory and matrix remodeling processes in both diseases

To investigate the potential shared functional pathways and regulatory mechanisms between OSCC and PD, we conducted GO and KEGG enrichment analyses. The intersection of DEGs identified from both OSCC and PD, as well as genes from the WGCNA modules, yielded 33 common genes (Fig. 3A). These genes were subjected to enrichment analysis to uncover their involvement in biological processes (BP), cellular components (CC), and molecular functions (MF). GO analysis revealed that the biological processes were primarily associated with extracellular matrix organization, extracellular structure organization, and external encapsulating structure organization (Fig. 3B). These results suggest that ECM-related genes are consistently altered in both diseases, implying that ECM remodeling is a shared pathological feature. Cellular components were predominantly localized to collagen-containing extracellular matrix and basement membrane, while molecular functions were significantly related to extracellular matrix structural constituents and collagen binding (Fig. 3B). These findings suggest that extracellular matrix remodeling may be a common pathological feature in both OSCC and PD, potentially contributing to disease progression and severity. The results of the KEGG enrichment analysis indicate that the primary pathways involved are the IL-17 signaling pathway and the Relaxin signaling pathway (Fig. 3C). This indicates that inflammation-related signaling may also be a converging mechanism linking OSCC and PD pathogenesis.

### Key biomarkers identified in OSCC and PD using multiple machine learning algorithms

**Fig. 4:**
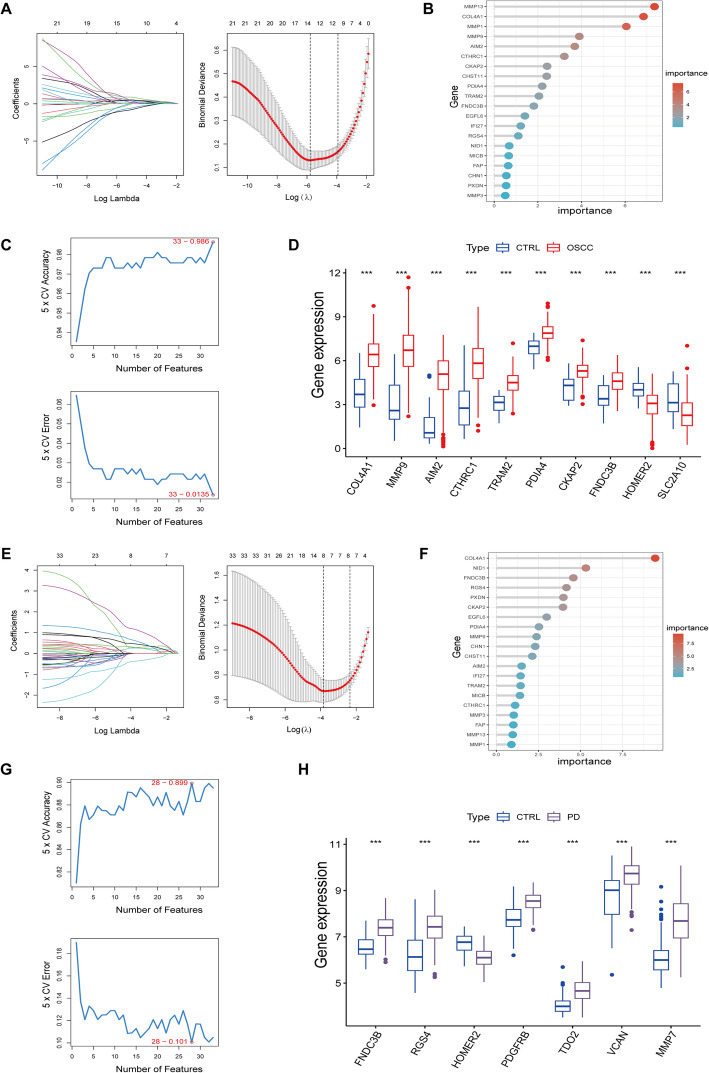
Machine Learning-Based Feature Gene Selection for OSCC and PD. **A**–**C** Feature gene screening in OSCC using Lasso regression, Random Forest (RF), and Support Vector Machine–Recursive Feature Elimination (SVM-RFE). **D** Boxplots showing the differential expression of the 10 OSCC feature genes (identified by the intersection of the three algorithms) between tumor and normal samples. **E**–**G**. Feature gene screening for PD using Lasso, RF, and SVM-RFE algorithms. **H** Boxplots showing the expression of the 7 PD feature genes across disease and control samples. These genes were prioritized as robust candidates due to their consistent selection by multiple algorithms and significant expression differences

To refine the identification of key genes associated with OSCC and PD, we applied three machine learning algorithms: Lasso regression, Random Forest (RF), and Support Vector Machine Recursive Feature Elimination (SVM-RFE). The 33 intersecting genes were first analyzed using Lasso regression, which identified 14 feature genes in OSCC and 8 in PD (Fig. 4A and Fig. 4E). Subsequently, RF analysis, which selects genes based on importance scores greater than 1, yielded 17 feature genes for OSCC and 30 for PD (Fig. 4B and Fig. 4F). SVM-RFE was then employed to optimize feature selection, achieving maximum accuracy with 33 genes for OSCC and 28 for PD (Fig. 4C and Fig. 4G). The intersection of feature genes across all three methods resulted in 10 key genes for OSCC and 7 for PD. Among these, COL4A1, MMP9, AIM2, CTHRC1, TRAM2, PDIA4, CKAP2, and FNDC3B were upregulated in OSCC, while HOMER2 and SLC2A10 were downregulated (Fig. 4D). In PD, FNDC3B, RGS4, PDGFRB, TDO2, VCAN, and MMP7 were upregulated, with HOMER2 downregulated (Fig. 4H). The consistent selection of these genes across multiple algorithms suggests their strong discriminatory power and potential biological relevance. Among them, MMP9 and COL4A1, which are both involved in matrix remodeling, were specifically upregulated in OSCC, consistent with the enrichment analysis findings. These genes represent potential biomarkers or therapeutic targets, as they are consistently identified across multiple machine learning approaches, underscoring their significance in OSCC and PD pathophysiology.

### Feature gene validation and risk prediction via nomogram models

**Fig. 5:**
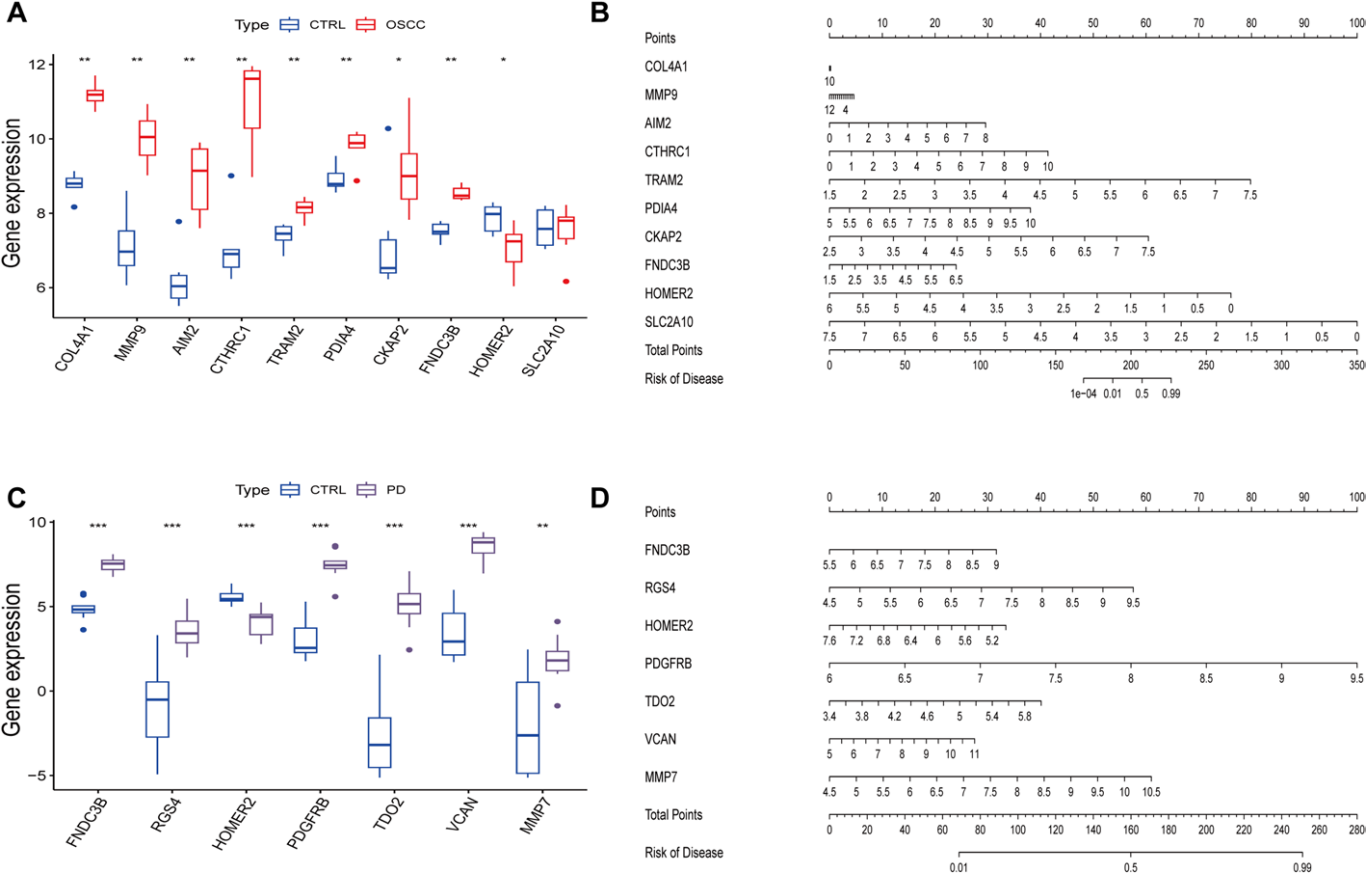
Nomogram Analyses and Feature Gene Validation in OSCC and PD. **A** Validation of OSCC key gene expression in the GSE246050 dataset. Eight genes were significantly upregulated and one downregulated. **B** Nomogram model for OSCC constructed using validated genes, illustrating the contribution of each gene to disease risk prediction. **C** Validation of PD feature genes in the GSE223924 dataset, confirming differential expression patterns. **D** Nomogram model for PD, enabling individualized risk estimation

To validate the identified feature genes, we employed nomogram analysis using independent datasets, GSE246050 for OSCC and GSE10334 for PD. After standardizing the data, the expression levels of the selected feature genes were reanalyzed. The results confirmed that in OSCC, COL4A1, MMP9, AIM2, CTHRC1, TRAM2, PDIA4, CKAP2, and FNDC3B were significantly upregulated, while HOMER2 was downregulated (Fig. 5A). However, SLC2A10 did not show a significant differential expression. In PD, FNDC3B, RGS4, PDGFRB, TDO2, VCAN, and MMP7 were upregulated, and HOMER2 was downregulated (Fig. 5C). These genes were used to construct nomograms, which predict the probability of disease based on gene expression profiles. The nomograms for OSCC and PD, illustrated in Fig. 5B and D, respectively, provide a visual tool for assessing the risk of disease based on the expression of these key genes. To evaluate the predictive performance of the nomogram models, we further conducted calibration curve and decision curve analyses. The calibration curves demonstrated good agreement between predicted and observed outcomes for both OSCC (Supplementary Fig. 2A) and PD (Supplementary Fig. 2C). Additionally, decision curve analysis (DCA) indicated favorable clinical net benefit across a range of threshold probabilities for OSCC (Supplementary Fig. 2B) and PD (Supplementary Fig. 2D), supporting the potential clinical utility of the nomogram models.

### Distinct immune cell infiltration and ECM signaling activation in OSCC and PD

**Fig. 6:**
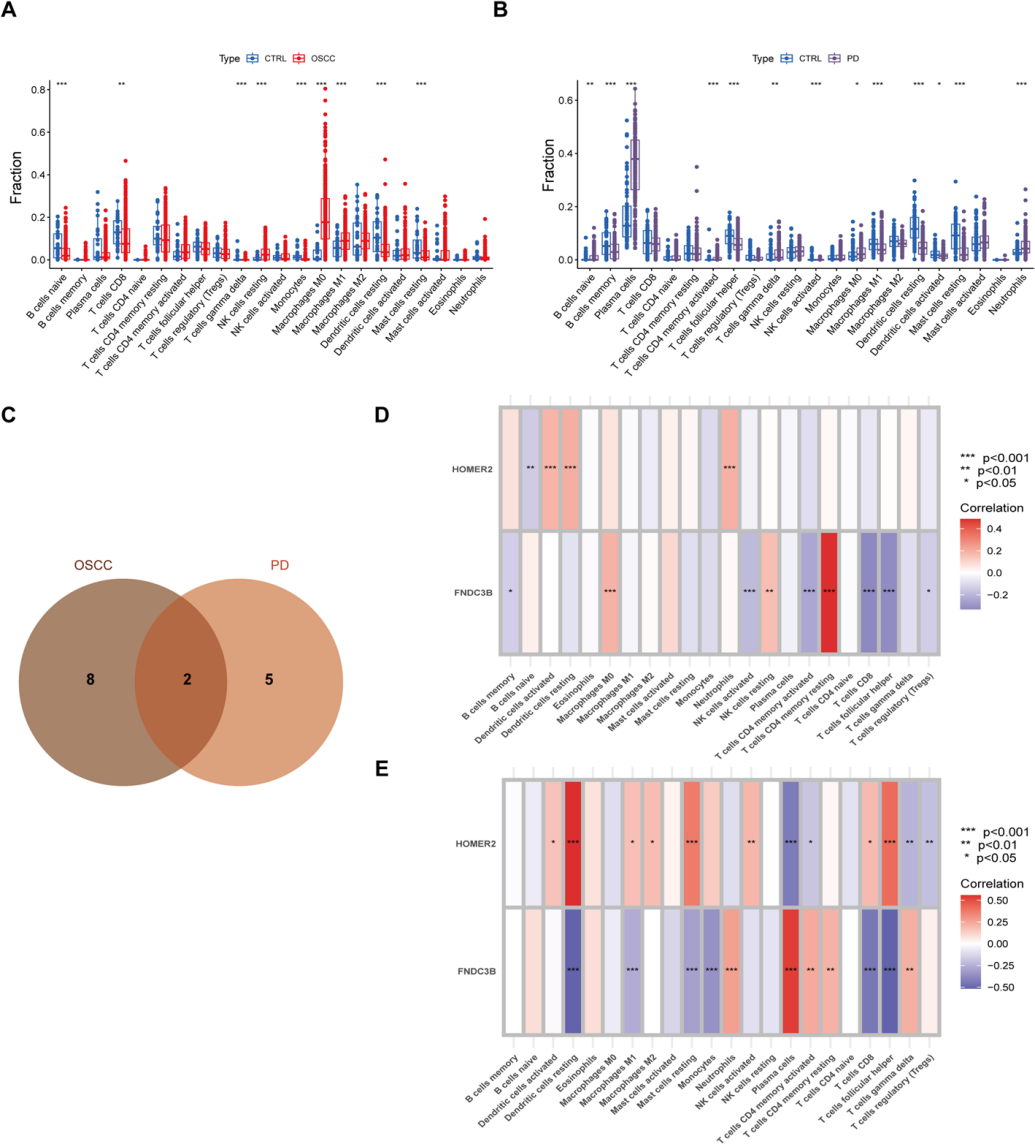
Immune Infiltration Analysis and Venn Diagram of OSCC and PD Feature Genes. **A**–**B** Analysis of immune cell infiltration in OSCC and PD samples using CIBERSORT. Significant differences in immune cell composition were observed between disease and control groups. **C** Venn diagram showing the intersection of feature genes identified in OSCC and PD. **D**–**E** Spearman correlation analysis between FNDC3B, HOMER2, and immune cell infiltration levels in OSCC and PD

To explore the immune landscape of OSCC and PD, we performed immune infiltration analysis using the CIBERSORT algorithm. This method estimates the relative proportions of 22 immune cell types within the transcriptomic data from OSCC and PD samples. The differences in immune cell infiltration between disease and normal groups were statistically assessed, as shown in Fig. 6A and B. Notably, OSCC tissues displayed increased infiltration of macrophages, while PD samples exhibited elevated levels of plasma cells and Neutrophils, reflecting disease-specific immune microenvironments. We then conducted a Spearman correlation analysis to evaluate the relationship between the expression levels of the intersecting feature genes, FNDC3B and HOMER2 (Fig. 6C), and the infiltration levels of these immune cells. In OSCC, HOMER2 was positively correlated with dendritic cells and neutrophils, but negatively correlated with naïve B cells. FNDC3B showed a positive correlation with M0 macrophages and a negative correlation with NK cells (Fig. 6D). In PD, HOMER2 was positively correlated with dendritic cells and negatively correlated with plasma cells, whereas FNDC3B was positively correlated with plasma cells and CD4 + T cells but negatively correlated with dendritic cells and M1 macrophages (Fig. 6E). These associations suggest that HOMER2 and FNDC3B may play divergent roles in modulating immune responses depending on the disease context. Furthermore, GSEA revealed that FNDC3B was positively associated with the ECM receptor interaction pathway in both OSCC and PD, highlighting a potential shared signaling mechanism across these conditions (S. Fig. 2A and B). This finding aligns with the ECM remodeling signatures observed in earlier analyses and suggests that FNDC3B may serve as a key mediator linking immune infiltration and extracellular matrix dynamics in both diseases.

### Cell-type-specific expression and ECM interactions revealed by single-cell transcriptomics

**Fig. 7:**
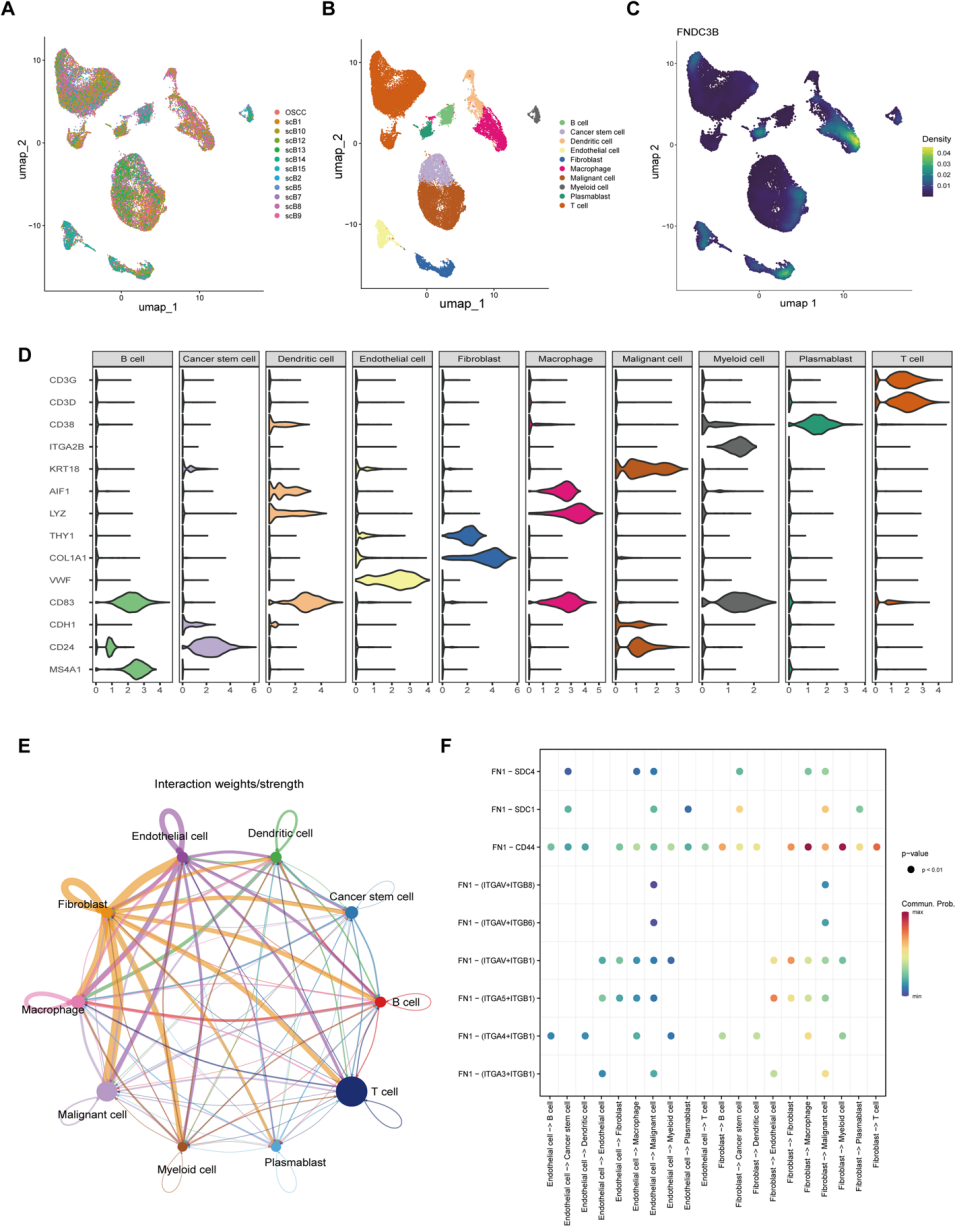
Single-Cell RNA Sequencing Analysis of OSCC. ** A** UMAP plot of integrated OSCC single-cell data after batch effect removal using Harmony, revealing clear separation into major cell populations. **B** UMAP plot showing the clustering of OSCC cells into 10 distinct cell types. **C** Feature plot illustrating FNDC3B expression predominantly in macrophages within OSCC. **D** Violin plots of marker genes used to annotate the cell clusters identified in OSCC. **E** Cell–cell interaction network among OSCC clusters based on inferred ligand–receptor communication. **F** Detailed view of FN1 protein interactions with its binding partners in the extracellular matrix in OSCC

Single-cell RNA sequencing data from OSCC and PD tissues underwent quality control, dimensionality reduction, and clustering. Figure 7A shows the UMAP projection of OSCC cells after batch effect correction using Harmony, revealing well-separated clusters with distinct transcriptional identities. In OSCC, the cells were classified into 10 distinct clusters, including T cells (CD3D, CD3G), malignant cells (CDH1, KRT18), macrophages (AIF1, LYZ), cancer stem cells (CD24, CDH1), fibroblasts (COL1A1, THY1), B cells (MS4A1), endothelial cells (VWF), dendritic cells (CD83), plasmablasts (CD38), and myeloid cells (ITGA2B) (Fig. 7B). FNDC3B was predominantly enriched in macrophages (Fig. 7C). This suggests that FNDC3B may participate in immune-related functions or inflammatory signaling within the tumor microenvironment. The violin plots in Fig. 7D display the marker genes used for cell cluster annotation, while Fig. 7E illustrates the cell-cell interaction network among the 10 clusters. Figure 7F highlights the interaction between FN1 protein and its binding partners within the extracellular matrix, including FN1-SDC4, FN1-SDC1, FN1-CD44, and FN1-integrin subunits. These interactions indicate potential roles in cell adhesion and matrix remodeling that could influence tumor progression.

For PD, Supplementary Fig. 3A displays the Harmony-integrated UMAP distribution of PD cells, revealing clear delineation of 10 cellular populations following batch correction. These clusters cells were similarly classified into 10 clusters: T cells (CD3D, CD3G), epithelial cells (KRT13), basal cells (LGALS3), NK cells (KLRD1), mesenchymal stem cells (CD44), endothelial cells (ECSCR, VWF), B cells (MS4A1, CD19), macrophages (LYZ, AIF1), mesenchymal cells (COL1A2), and multipotent progenitor cells (ISL1) (S. Figure 3B). FNDC3B was primarily enriched in endothelial cells (S. Figure 3C). The marker gene violin plots for these clusters are shown in Supplementary Fig. 3D, with Supplementary Fig. 3E depicting the cell-cell interaction network. Supplementary Fig. 3F demonstrates the extent of interaction between FN1 protein and its partners in the extracellular matrix, including FN1-SDC4, FN1-SDC1, FN1-CD44, and FN1-integrin subunits. Similar interaction patterns in both diseases imply that extracellular matrix dynamics and FN1-mediated signaling might represent a shared regulatory axis in OSCC and PD pathogenesis.

## Discussion

OSCC and PD are two distinct yet increasingly recognized conditions within oral health, each with unique pathophysiological characteristics. OSCC, a malignancy of the oral epithelium, is primarily driven by genetic mutations, chronic inflammation, and alterations in cellular signaling pathways, often leading to aggressive tumor progression and poor prognosis [22–24]. Conversely, PD is a chronic inflammatory disorder affecting the supporting structures of the teeth, primarily caused by bacterial infection and characterized by the destruction of periodontal tissues [25, 26]. Despite their disparate natures, emerging evidence suggests that chronic inflammatory conditions like PD might contribute to an increased risk of OSCC development [27, 28]. This is supported by studies indicating that chronic inflammation can create a microenvironment conducive to malignant transformation [29–31]. Our study aimed to elucidate potential molecular and immune-related commonalities between OSCC and PD by analyzing differential gene expression and identifying shared regulatory mechanisms.

By performing enrichment analyses, we observed significant involvement of ECM remodeling pathways in both OSCC and PD. The GO analysis highlighted key biological processes such as extracellular matrix organization, extracellular structure organization, and external encapsulating structure organization, aligning with previous findings that ECM remodeling is a hallmark of cancer progression and tissue inflammation [32–36]. Additionally, GSEA supported these findings by showing that FNDC3B, a gene associated with ECM interactions, was positively correlated with this pathway in both OSCC and PD. This suggests that ECM dysregulation might be a shared mechanism contributing to disease pathology. FNDC3B has been implicated in regulating extracellular matrix composition and interacting with key partners such as integrins and syndecans, which can influence not only cell migration but also immune cell behavior [37–39]. In both OSCC and PD, such interactions may mediate immune cell recruitment or exclusion, as well as impact the structural remodeling of affected tissues. The role of ECM in tumor progression and chronic inflammation has been well-documented, reinforcing the relevance of these findings.

The immune infiltration analysis revealed distinct correlations between feature genes and immune cell types in OSCC and PD. In OSCC, HOMER2, a gene involved in synaptic signaling and immune regulation, was positively correlated with dendritic cells and neutrophils but negatively with naïve B cells [40, 41]. FNDC3B, known for its role in extracellular matrix organization and cellular signaling, was positively associated with M0 macrophages and negatively with NK cells [37, 42, 43]. This finding is consistent with the notion that OSCC tumors exhibit a complex immune landscape with varying immune cell interactions influencing tumor progression [44–46]. In PD, HOMER2 correlated positively with dendritic cells and negatively with plasma cells, whereas FNDC3B was positively correlated with plasma cells and CD4 + T cells, and negatively with dendritic cells and M1 macrophages. These differences raise the possibility that targeting FNDC3B could differentially modulate the immune microenvironment and ECM remodeling in OSCC and PD. Further studies exploring downstream signaling pathways, such as TGF-β, PI3K/Akt, or AMPK pathway, may help elucidate the mechanistic basis of FNDC3B’s dual role in these conditions [47–49].

This study provides a comprehensive analysis of the shared molecular and immune features between OSCC and PD, revealing commonalities in ECM remodeling and immune infiltration patterns. The identification of FNDC3B as a key gene associated with ECM-receptor interaction in both conditions underscores its potential as a therapeutic target and biomarker for disease progression. However, the study has limitations, including the reliance on publicly available datasets, which may introduce variability not present in controlled experimental settings. Additionally, the study’s cross-sectional nature limits causal inferences regarding the relationship between PD and OSCC. Future work should also consider integrating research from tumor immunology and matrix biology to strengthen the mechanistic context of our findings. Emphasizing the translational relevance of these insights could improve our understanding of how FNDC3B contributes to disease progression and its potential as a targetable molecule in clinical settings. Integrating these findings into clinical frameworks may support improved early detection and intervention strategies for both OSCC and PD.

## Conclusion

This study elucidates key molecular insights into OSCC and PD, revealing both shared and distinct features between these diseases. Our analysis identified common pathways, particularly ECM-receptor interactions, and highlighted FNDC3B as a critical gene implicated in both conditions. The application of machine learning methods further refined the identification of potential biomarkers. While these findings lay a foundation for understanding disease mechanisms and developing new interventions, future research with experimental validation is essential to confirm these results and explore their clinical implications.

## Supplementary Information

Below is the link to the electronic supplementary material.

Supplementary Material 1.

Supplementary Material 2. Figure. S1. DEGs and WGCNA Analysis of PD. (A) Volcano plot showing differentially expressed genes between 183 PD samples and 64 normal tissue samples. Red and green dots represent significantly upregulated and downregulated genes, respectively (|log2FC| >0.5, adjusted p-value < 0.05). (B) Scale-free topology analysis plot identifying power = 16 as optimal for PD co-expression network construction using WGCNA. (C) Hierarchical clustering dendrogram of genes in PD, with modules identified using dynamic tree cutting. (D) Heatmap of module–trait correlations in PD, based on Spearman coefficients. Four modules (MEblack, MEblue, MEgrey, MEred) were highly correlated with disease status and selected for downstream functional enrichment analysis.

Supplementary Material 3. Fig. S2. Evaluation of Predictive Performance of Nomogram Models for OSCC and PD. (A) Calibration curve for OSCC, showing close alignment between predicted and actual outcomes, indicating high model accuracy. (B) Decision curve analysis (DCA) for OSCC, demonstrating a positive net clinical benefit across a wide range of threshold probabilities. (C) Calibration curve for PD, similarly displaying good consistency between predicted probabilities and observed clinical outcomes. (D) DCA for PD, confirming the practical value of the nomogram in clinical decision-making scenarios by showing robust net benefit.

Supplementary Material 4. Figure. S3. GSEA Analysis of OSCC and PD. (A-B) GSEA results showing the association of FNDC3B with the ECM receptor interaction pathway in both OSCC (A) and PD (B).

Supplementary Material 5. Figure. 4. Single-Cell RNA Sequencing Analysis of PD. (A) UMAP plot of integrated PD single-cell data after batch effect correction using Harmony. (B) UMAP plot showing the clustering of PD cells into 10 distinct cell types. (C) Feature plot illustrating FNDC3B expression predominantly in endothelial cells within PD. (D) Violin plots of marker genes used to annotate the cell clusters identified in PD. (E) Cell-cell interaction network among PD clusters. (F) Detailed view of FN1 protein interactions with its binding partners in the extracellular matrix in PD.

## Data Availability

Transcriptomic and clinical data for oral cancer were obtained from TCGA (https://portal.gdc.cancer.gov/projects/TCGA-HNSC). Datasets for oral cancer and periodontitis (GSE10334, GSE246050, GSE223924, GSE215403, GSE266897) were downloaded from GEO (https://www.ncbi.nlm.nih.gov/geo/). All original data are included in the article and Supplementary Material. Further inquiries can be directed to the corresponding authors.
