# Supplementary figures and images for: Integrated bioinformatics analysis of common molecular mechanisms and biomarkers in oral squamous cell carcinoma and periodontal disease
Source: Discov Oncol. 2025 Oct 15;16:1891. doi: 10.1007/s12672-025-03728-0 (PMC12528526; doi:10.1007/s12672-025-03728-0)

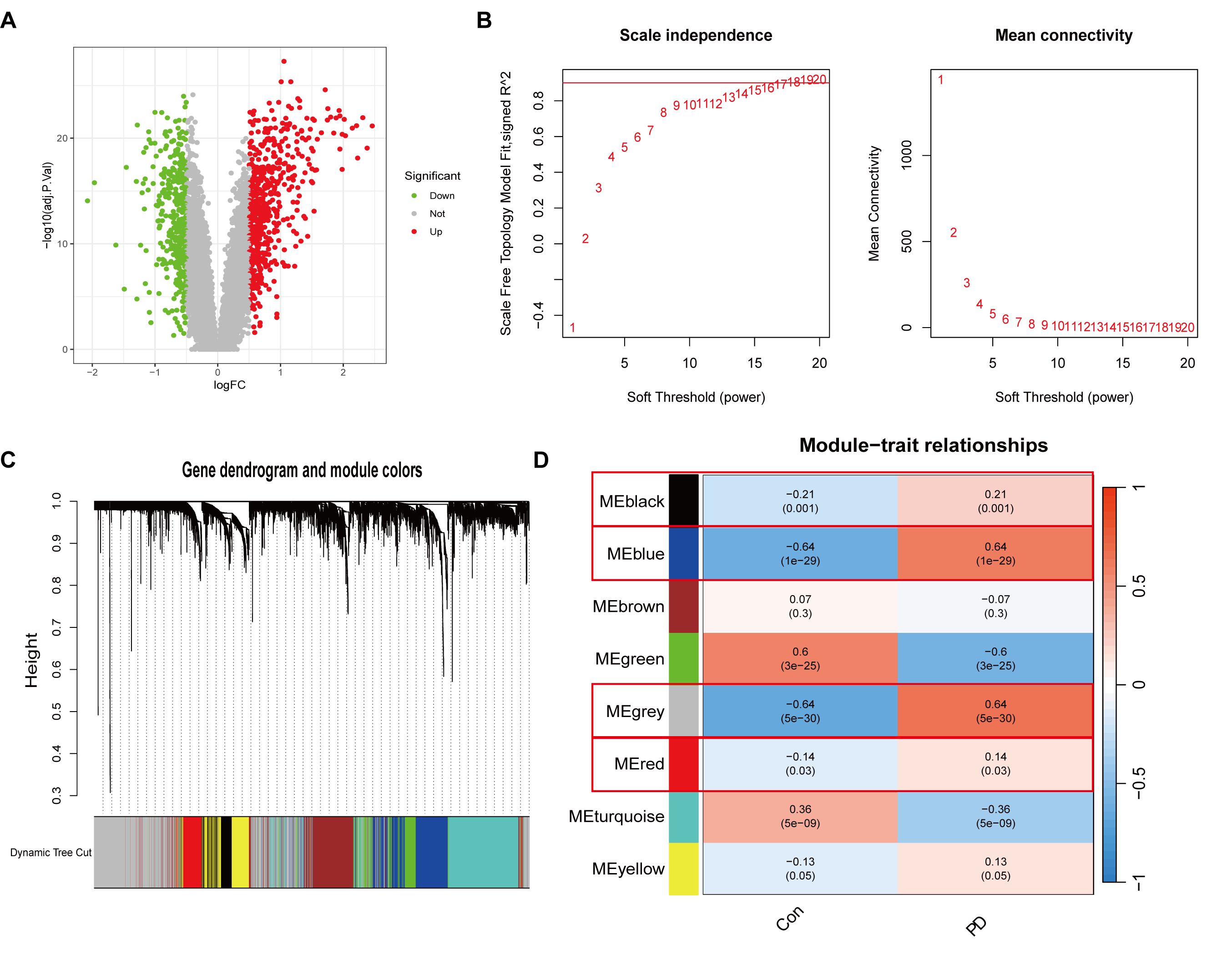

Supplement: Supplementary file 2 — Supplementary Material 2. Figure. S1. DEGs and WGCNA Analysis of PD. (A) Volcano plot showing differentially expressed genes between 183 PD samples and 64 normal tissue samples. Red and green dots represent significantly upregulated and downregulated genes, respectively (|log2FC| >0.5, adjusted p-value < 0.05). (B) Scale-free topology analysis plot identifying power = 16 as optimal for PD co-expression network construction using WGCNA. (C) Hierarchical clustering dendrogram of genes in PD, with modules identified using dynamic tree cutting. (D) Heatmap of module–trait correlations in PD, based on Spearman coefficients. Four modules (MEblack, MEblue, MEgrey, MEred) were highly correlated with disease status and selected for downstream functional enrichment analysis. [file 12672_2025_3728_MOESM2_ESM.tif]

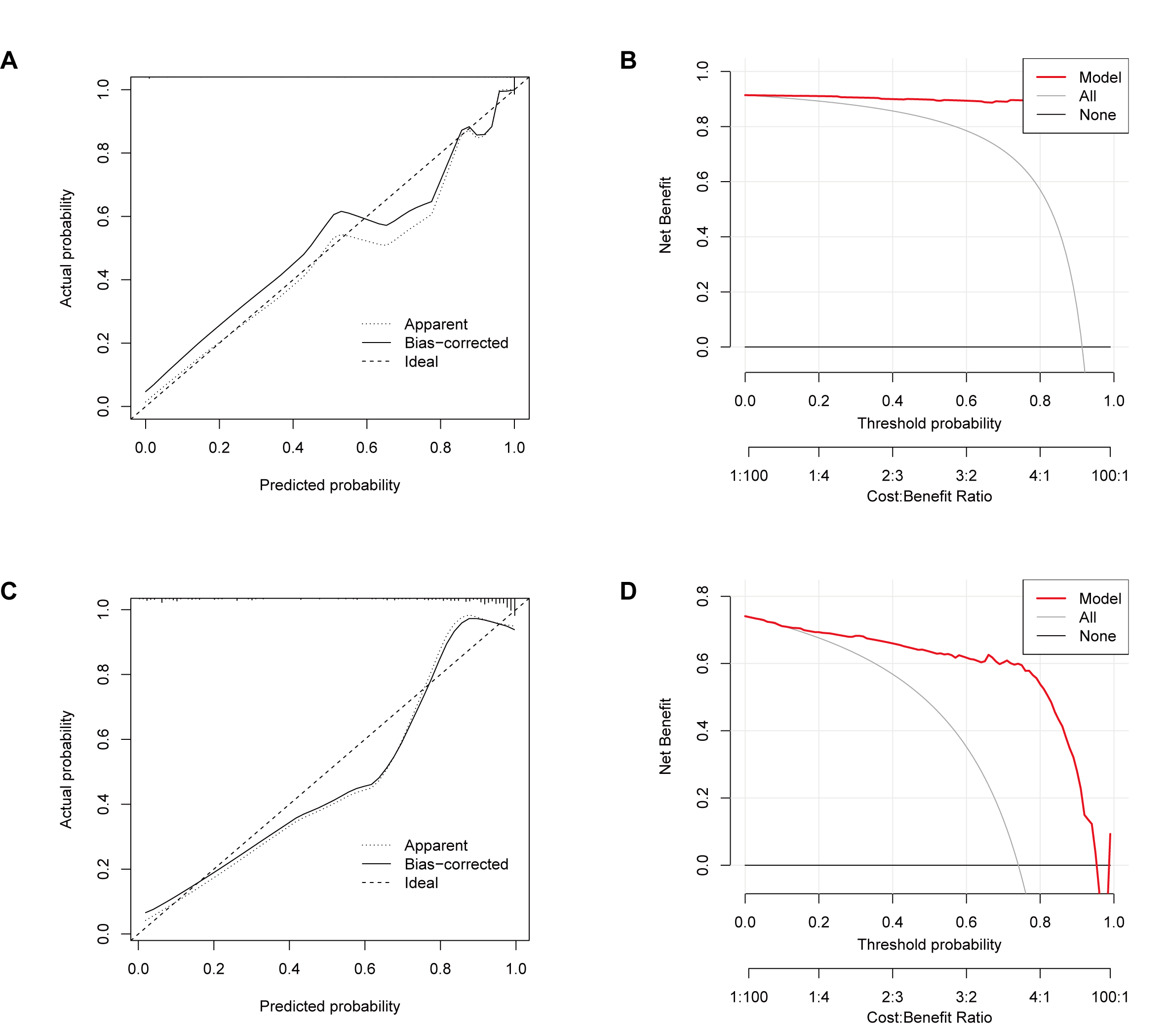

Supplement: Supplementary file 3 — Supplementary Material 3. Fig. S2. Evaluation of Predictive Performance of Nomogram Models for OSCC and PD. (A) Calibration curve for OSCC, showing close alignment between predicted and actual outcomes, indicating high model accuracy. (B) Decision curve analysis (DCA) for OSCC, demonstrating a positive net clinical benefit across a wide range of threshold probabilities. (C) Calibration curve for PD, similarly displaying good consistency between predicted probabilities and observed clinical outcomes. (D) DCA for PD, confirming the practical value of the nomogram in clinical decision-making scenarios by showing robust net benefit. [file 12672_2025_3728_MOESM3_ESM.tif]

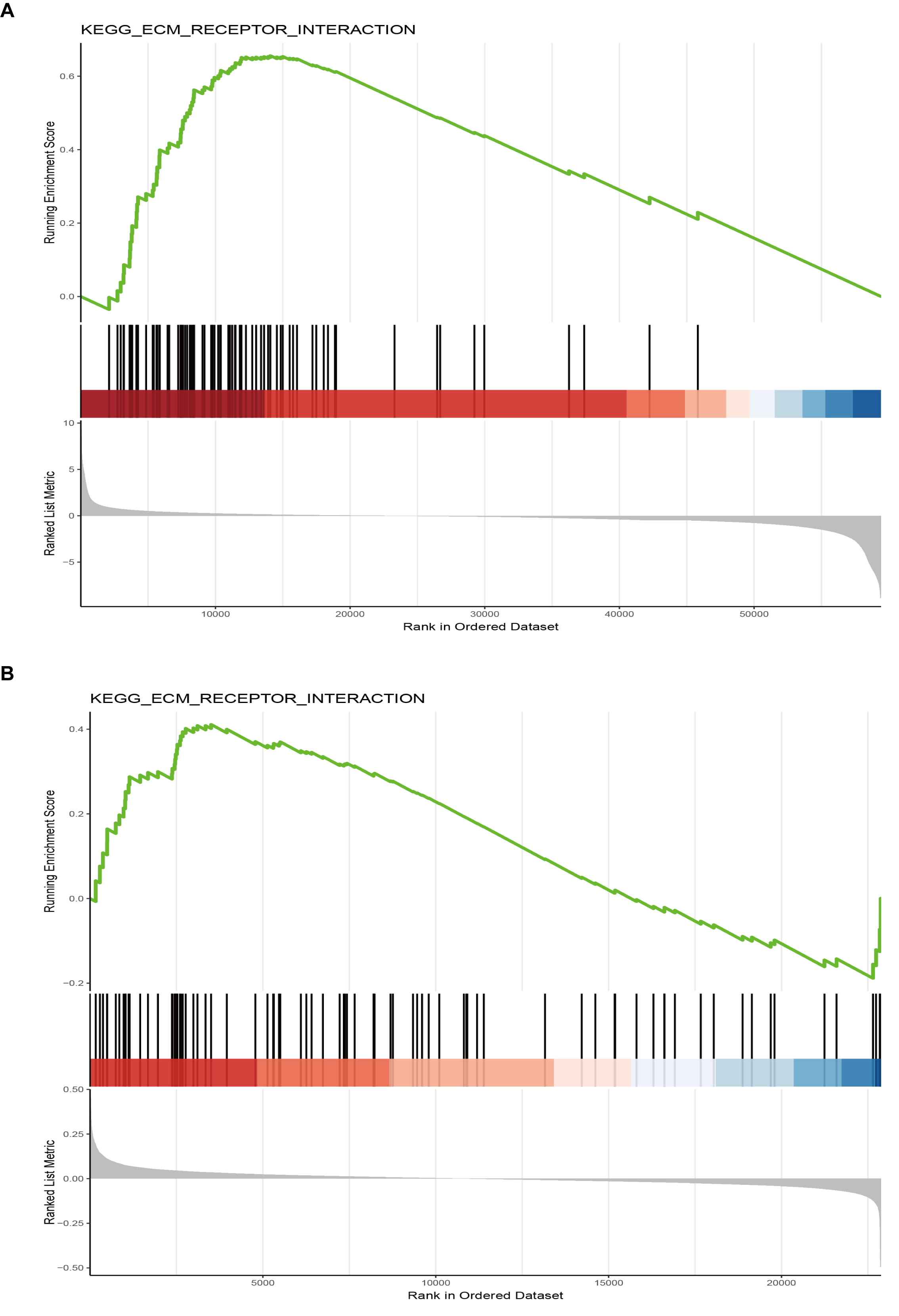

Supplement: Supplementary file 4 — Supplementary Material 4. Figure. S3. GSEA Analysis of OSCC and PD. (A-B) GSEA results showing the association of FNDC3B with the ECM receptor interaction pathway in both OSCC (A) and PD (B). [file 12672_2025_3728_MOESM4_ESM.tif]

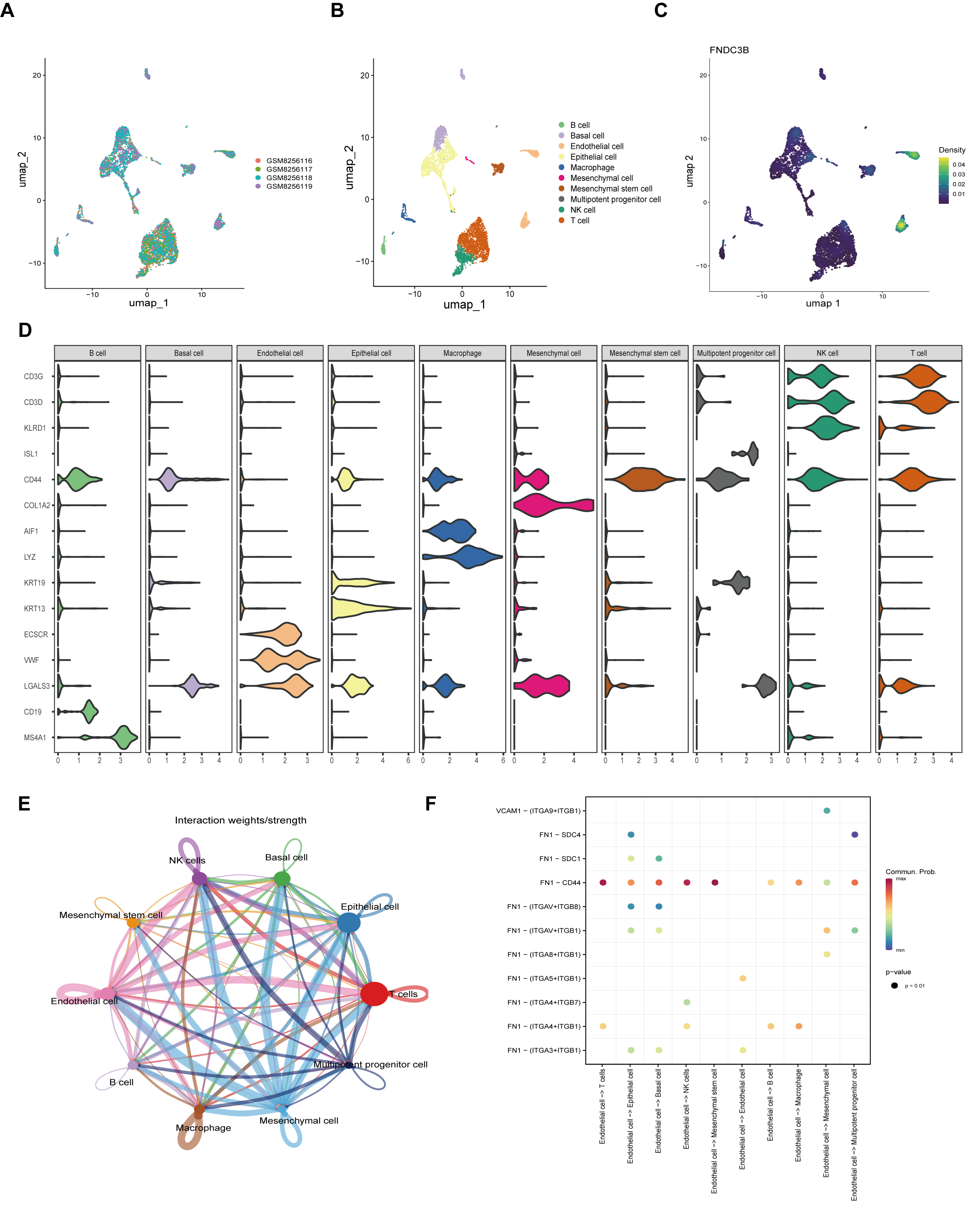

Supplement: Supplementary file 5 — Supplementary Material 5. Figure. 4. Single-Cell RNA Sequencing Analysis of PD. (A) UMAP plot of integrated PD single-cell data after batch effect correction using Harmony. (B) UMAP plot showing the clustering of PD cells into 10 distinct cell types. (C) Feature plot illustrating FNDC3B expression predominantly in endothelial cells within PD. (D) Violin plots of marker genes used to annotate the cell clusters identified in PD. (E) Cell-cell interaction network among PD clusters. (F) Detailed view of FN1 protein interactions with its binding partners in the extracellular matrix in PD. [file 12672_2025_3728_MOESM5_ESM.tif]
